# Robust increase in observed heat storage by the global subsurface

**DOI:** 10.1126/sciadv.adw9958

**Published:** 2025-11-12

**Authors:** Francisco José Cuesta-Valero, Almudena García-García, Hugo Beltrami, Félix García-Pereira, J. Fidel González-Rouco, Jian Peng

**Affiliations:** ^1^ Department of Remote Sensing, Helmholtz Centre for Environmental Research–UFZ, Leipzig 04318, Germany.; ^2^ Institute for Earth System Science and Remote Sensing, Leipzig University, Leipzig 04103, Germany.; ^3^ Climate & Atmospheric Sciences Institute, St. Francis Xavier University, Antigonish B2G 2W5, Canada.; ^4^ Faculty of Physical Sciences, Complutense University of Madrid, Madrid 28040, Spain.; ^5^ Instituto de Geociencias (IGEO, CSIC-UCM), Consejo Superior de Investigaciones Científicas, Madrid 28040, Spain.

## Abstract

Changes in heat storage within the different components of the climate system alter physical and biogeochemical phenomena relevant for human societies and ecosystems. Among such processes, permafrost thawing, soil carbon storage, and surface energy exchanges depend on the persistent heat gain by the continental subsurface. Nevertheless, there are not enough data to estimate ground heat storage at the global scale after the year 2000. We solve this problem by expanding the database of geothermal data with remote sensing observations from satellite platforms. Estimates from satellite data show a heat gain between 16.4 ± 3.4 and 21.78 ± 0.62 zettajoules during the past six decades. The global ground heat storage presents a positive acceleration between 0.16 ± 0.15 and 0.624 ± 0.032 zettajoules per square decade, similarly to the rest of components of the Earth heat inventory. The planned satellite missions ensure the monitoring of the land component of the Earth heat inventory in the future.

## Teaser

Global ground heat storage from satellite, geothermal, and meteorological data shows an accelerated increase in recent decades.

## INTRODUCTION

The Earth heat inventory is a fundamental indicator of the evolution of ongoing climatic change (*1*–*3*). It consists of observations of heat storage evolution in the ocean, the land surface and subsurface, the cryosphere, and the atmosphere in response to the radiative imbalance at the top of the atmosphere (*4*). If the radiative imbalance is positive, this leads to an increase in the total heat storage in the Earth system, whereas a negative imbalance leads to a decrease in total heat. In a stable climate, the radiative imbalance is zero over long timescales, resulting in no net heat gain within the Earth system. The determination of this radiative imbalance from satellite data at the top of the atmosphere is difficult because the imbalance is markedly smaller than the magnitude of the fluxes involved in the calculations: the incoming and reflected solar radiation and emitted longwave radiation into space (*5*). The alternative approach consists of quantifying and monitoring the heat storage in all climate subsystems, complementing the imbalance estimates from satellite data with measurements of the total heat storage in the climate system (*6*).

Although the ocean is the largest component of the Earth heat inventory, the monitoring of the smaller terms is necessary to obtain the full picture of what is occurring in the climate system, as well as to distinguish the response to the anthropogenic forcing from changes associated with internal variability that can also impact the energy balance (*7*). In addition, the heat allocated in the smaller components of the Earth heat inventory influences physical processes that are crucial for both society and ecosystems. This can happen through increasing the temperature of a subsystem, e.g., a warmer atmosphere can transport higher amounts of water vapor, intensifying extreme precipitation events (*8*). In turn, changes associated with heat gain are not necessarily the result of a local temperature increase and can also drive phase changes in the cryosphere, such as glacier melting, which further contributes to rising global sea level (*9*).

Ground heat storage (GHS) is the second-largest term of the Earth heat inventory storing 5 to 6% of the total heat (*1*, *3*, *10*, *11*). Increases in GHS warm the subsurface and trigger permafrost thawing at high latitudes of the Northern Hemisphere (*12*, *13*), activating the permafrost carbon feedback (*14*, *15*), and enhancing the projected global warming by the end of the century (*16*), therefore hindering efforts to accomplish the temperature targets of the Paris Agreement (*17*). Furthermore, the warming of the shallow subsurface contributes to intensifying extreme heat under dry soil conditions (*18*) and can alter the diversity of microbial and fungal communities within the soil (*19*).

Previous efforts have been made to quantify GHS from in situ geothermal data (*10*, *20*–*22*) and used to assess the realism of global climate simulations (*23*–*28*). Furthermore, some studies have substituted the temporally limited geothermal data with meteorological observations and reanalyses (*27*, *29*), therefore offering updated estimates of GHS. The resulting estimates show uncertainties that likely arise from methodological and data availability limitations.

Here, we assess GHS using observational data from 1960 to 2020 and present a previously unavailable estimate derived from satellite data. We analyze GHS from geothermal (*30*), meteorological (*27*), and satellite estimates together. We also combine satellite and geothermal data to obtain a continuous GHS estimate for the entire period of interest. The rate of change of GHS is then evaluated in the context of the Earth heat inventory.

**Fig. 1.**
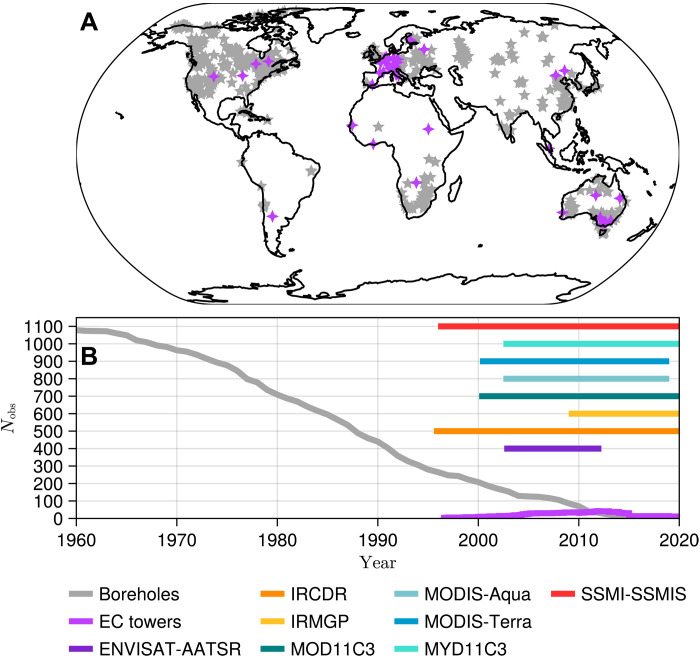
Availability of geothermal, eddy-covariance, and satellite data. Spatial (**A**) and temporal (**B**) coverage of different datasets available for estimating GHS around the world. Gray stars represent geothermal data, purple stars represent eddy-covariance towers fulfilling the requirements detailed in the Materials and Methods section, and horizontal bars represent the temporal coverage of the different satellite LST products considered here. Satellite products have global coverage at monthly resolution, but areas below 60°S and over Greenland were removed in this work (see Materials and Methods).

Observing the land term of the Earth heat inventory is a complex task due to the lack of an adequate monitoring network that samples temperatures to several meters of depth in the terrestrial subsurface (*18*, *31*, *32*). Long-term estimates of GHS have previously been derived from inversions of geothermal data, specifically from subsurface temperature profiles at sufficient depth (*20*). Such a database has provided global estimates of ground heat flux (GHF) at the global scale since the year 1600 (*20*, *21*). Temperature and flux estimates from geothermal data only provide long-term changes in surface conditions, typically at multidecadal to centennial resolutions (*33*). Moreover, these data were mostly collected from boreholes of opportunity drilled as part of prospective campaigns by mining companies between the 1960s and 1980s, with the number of available observations rapidly decreasing since then and with large geographical gaps in the existing database (Fig. 1). The paucity in the number of temperature-depth profiles with time impinges on the robustness of global GHS estimates after the year 2000. Additional observational sources of information are necessary to expand the geothermal dataset and to derive a comprehensive estimate of global GHS that extends through the period 2000 to 2020.

Measurements of GHF from eddy-covariance towers (see Materials and Methods) are potential candidates for estimating heat storage. These observations are usually collected at half-hourly or hourly resolutions, thus greatly improving the temporal resolution of geothermal estimates. Nevertheless, spatial gaps in eddy-covariance data are larger than those in the geothermal dataset, and the number of towers with long enough records to be used in climate analyses is also low (around 20 stations in 2020; Fig. 1). Thus, eddy-covariance stations can complement estimates of heat storage for 2000 to 2020 but cannot produce a robust long-term global average.

Meteorological observations of surface air temperatures from a global gridded product have previously been used to derive GHF estimates in combination with a solution of the one-dimensional heat diffusion equation (*29*). Furthermore, a recent work estimates GHS (*27*) by propagating the global mean surface air temperature from six gridded observational datasets using a purely conductive forward model, generating three profiles per year representing the median and the uncertainty in the subsurface thermal properties. Once these profiles are integrated and multiplied by the continental area, a globally averaged heat storage estimate and its uncertainty was produced. Although this approach attempts to consider the plausible variations in global soil thermal properties associated with local soil heterogeneity, it does not accurately consider the effect of spatial changes in land cover types or changes in soil moisture when deriving the synthetic subsurface temperature profiles.

Remote sensing observations provide an interesting alternative for expanding global estimates of GHS from observations. Eight land surface temperature (LST) products covering all continental areas at monthly resolution are considered here. These products are developed from satellite remote sensing observations as part of the Climate Change Initiative of the European Space Agency (ESA-CCI) and NASA MODIS. These projects use measurements provided by sensors in satellites at different orbits around the Earth (Materials and Methods and table S1 in the Supplementary Materials). We use the satellite temperatures, combined with information about land cover type, soil moisture, and soil composition, to estimate GHFs at the land surface. Heat fluxes are typically derived from ground surface temperatures and the thermal inertia of the subsurface (*34*) (eq. S1). The thermal inertia consists in the ability of the ground to exchange heat with its surroundings, thus determining the heat propagation and storage. Nevertheless, satellite temperatures correspond to skin temperatures, which are correlated to ground surface temperatures but are not the same variable, and thermal inertia cannot be directly estimated from satellite observations. This thermal property is typically estimated as $\phi =\sqrt{\mathrm{\rho C}\cdot \lambda }$ , with ρC the volumetric heat capacity and λ the thermal conductivity of the medium, and can be derived from soil composition and soil moisture as in ref. (*35*). Therefore, although thermal inertia estimates for the ground are available from previous studies, satellite temperatures must be converted into ground temperatures before obtaining flux estimates. The main factor determining the difference between skin temperature and ground surface temperatures is the land coverage, i.e., forest, crops, urban areas, grasslands, and others (*36*). A solution for this problem was developed in ref. (*37*), which estimated an effective thermal inertia that accounts for both the physical properties of the ground and the land cover type at each location. Such effective thermal inertia captures the differences between skin temperatures and ground surface temperatures for every land cover type, allowing the use of satellite temperatures instead of ground surface temperatures to produce flux estimates. The effective thermal inertia is estimated here on the basis of ERA5-Land reanalysis data (see Materials and Methods). Furthermore, satellite flux estimates are combined with estimates from geothermal data to cover the period 1960 to 2020. Nevertheless, the differences in climatological conditions between the satellite period and preindustrial times should be reconciled. This step is necessary because the period of reference for geothermal data corresponds to approximately the 1300 to 1700 CE period and thus experienced different average temperature conditions than the near present. To this end, we follow two reconciliation approaches: a physical method based on expanding the temporal series of global average values from each satellite product by adding the global average of 1300 to 1700 CE from geothermal data at the beginning of the series and a statistical method based on adjusting the mean of the derived satellite fluxes to coincide with the mean of the geothermal estimates during the period of data overlap. The results of these approaches can be considered as an upper (statistical) and a lower (physical) estimates of the global GHS.

**Fig. 2.**
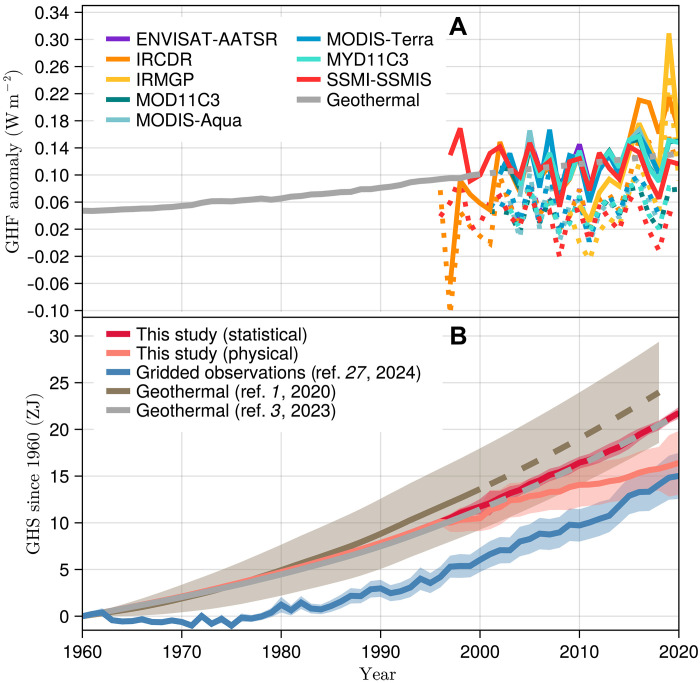
Temporal evolution of global GHF and global GHS. (**A**) GHF from geothermal data and satellite estimates. Dotted lines correspond to results from the physical reconciliation approach, whereas solid lines display results from the statistical reconciliation approach. (**B**) GHS from geothermal data, satellite estimates, and meteorological products. Satellite estimates of GHS consist of the mean (red lines) and the 95% confidence interval (red shadows) of an ensemble of products (see Materials and Methods). Results from refs. (*1*, *3*, *27*) are shown in brown, gray, and blue colors, respectively. The dashed lines indicate the extrapolated period.

Once the GHF is retrieved from each satellite product (Fig. 2A), heat storage is estimated by obtaining the cumulative sum of the heat fluxes with time. A global GHS temporal evolution is then created by combining the estimates from geothermal data from 1960 until the beginning of each satellite series (which ranges from 1995 to 2009; Fig. 1B and table S1), and continuing with satellite GHS estimates. To this end, the geothermal GHS value in the year before satellite records start is added to the satellite heat storage series to account for the heat stored before the satellite was launched. Thereby, an ensemble of eight GHS temporal series is generated from 1960 until the end of the satellite records.

## RESULTS

The combination of satellite and geothermal data confirms an increase in heat storage in the continental subsurface as shown in previous analyzes, yielding similar values to previous studies (Fig. 2) (*1*, *3*, *27*). Although the heat flux from Special Sensor Microwave Imager–Special Sensor Microwave Imager/Sounder (SSMI-SSMIS) data presents a negative trend (Fig. 2A), we keep it in the analysis as this is the only microwave product of the ensemble, and removing it does not change the main results (fig. S1). GHS between 1960 and 2018 reaches 15.8 ± 2.3 and 20.43 ± 0.52 ZJ considering the physical and statistical reconciliation approaches, respectively, with previous results yielding 24.0 ± 5.4 ZJ (*1*), 20.47 ± 0.19 ZJ (*3*), and 14.1 ± 2.2 ZJ (*27*). The result from ref. (*27*) was derived from gridded meteorological observations of surface air temperature, and it is consistent with the combined geothermal and satellite estimates obtained here (Fig. 2B) but presenting slightly lower values during all the period of interest. Such a difference probably arises because geothermal data include information about the transient state of the ground in the past, therefore yielding a higher heat uptake at the beginning of the period (*33*).

GHS since the year 1960 from combined satellite and geothermal data presents a global trend of 2.879 ± 0.047 ZJ dec^−1^ (*P* < 0.01, Mann-Kendall test) considering the physical reconciliation approach and 3.54 ± 0.19 ZJ dec^−1^ (*P* < 0.01) considering the statistical reconciliation approach. The trend from the statistical approach is similar to the trend extrapolated from geothermal data alone (3.52 ± 0.22 ZJ dec^−1^, *P* < 0.01) (*3*), with both physical and statistical estimates being higher than the ~2.70 ± 0.32 ZJ dec^−1^ (*P* < 0.01) trend from gridded observations (*27*). The extrapolation of the geothermal estimates of GHS yielded a higher heat storage trend after the year 2000, specifically 5.09 ± 0.11 ZJ dec^−1^ (*P* < 0.01), whereas both multisatellite averages yield a lower trend during the same period of time. The physical reconciliation approach achieves a trend of 2.47 ± 0.18 ZJ dec^−1^ (*P* < 0.01), whereas the statistical approach achieves 4.84 ± 0.17 ZJ dec^−1^ (*P* < 0.01) since the year 2000. The estimates from gridded observations present a trend of 4.41 ± 0.39 ZJ dec^−1^ (*P* < 0.01) for the same period, closer to satellite estimates than to geothermal estimates.

Heat storage estimates shown in Fig. 2 are generated using three different data sources combined with two different techniques, which introduces important methodological differences that should be considered for interpreting these results. GHS estimates from satellite and geothermal data are retrieved from surface temperature series and a solution of the one-dimensional heat diffusion equation (eq. S1) to derive changes in GHF at the terrestrial surface, with heat storage obtained as the cumulative sum of the changes in surface flux. Otherwise, GHS estimates from gridded observations of surface air temperatures were retrieved by propagating the changes in surface temperature through the ground (*27*) to generate synthetic subsurface temperature profiles, which were then integrated with depth to obtain the heat stored in the subsurface. Although there are differences in the factors analyzed to determine the required thermal properties by each method, both approaches lead to consistent estimates. Estimates from both geothermal data and gridded observations consider a range of probable values for the thermal inertia and thermal diffusivity of the ground, accounting only for the averaged mineral composition of the ground until depths of several hundreds of meters (*21*, *27*). These values are considered constant through the subsurface and contribute to the uncertainty provided in the results displayed here. Satellite estimates, otherwise, consider the spatial pattern of soil moisture, the land cover, and the mineral composition of the ground at each location to produce a global effective thermal inertia. Therefore, although the thermal inertia included in the satellite estimates constitutes a more realistic value at the surface, this value of thermal inertia cannot be used to estimate heat storage from geothermal data and gridded observations. This is because both geothermal data and gridded observations need information about the averaged thermal properties of the ground until depths of several hundreds of meters, but the values retrieved for the satellite estimates correspond to those of the terrestrial surface. Other important points to consider are the differences in temporal resolution and spatial coverage of the three GHS estimates. Although heat storage from satellite and gridded observation products achieve annual resolution, the geothermal data are only able to yield results for 30-year periods. This is a limitation imposed by the physics of heat propagation because the ground acts as a low-pass filter removing short-term variability in surface conditions and thus only recording low-frequency signals (*33*). Furthermore, the geothermal dataset presents a much more limited amount of data records and covers a smaller area than the satellite and gridded observation products (Fig. 1). Nevertheless, the logs in the geothermal data have been shown to represent global changes in surface temperature before the year 2000 in agreement with meteorological products (*21*).

**Fig. 3.**
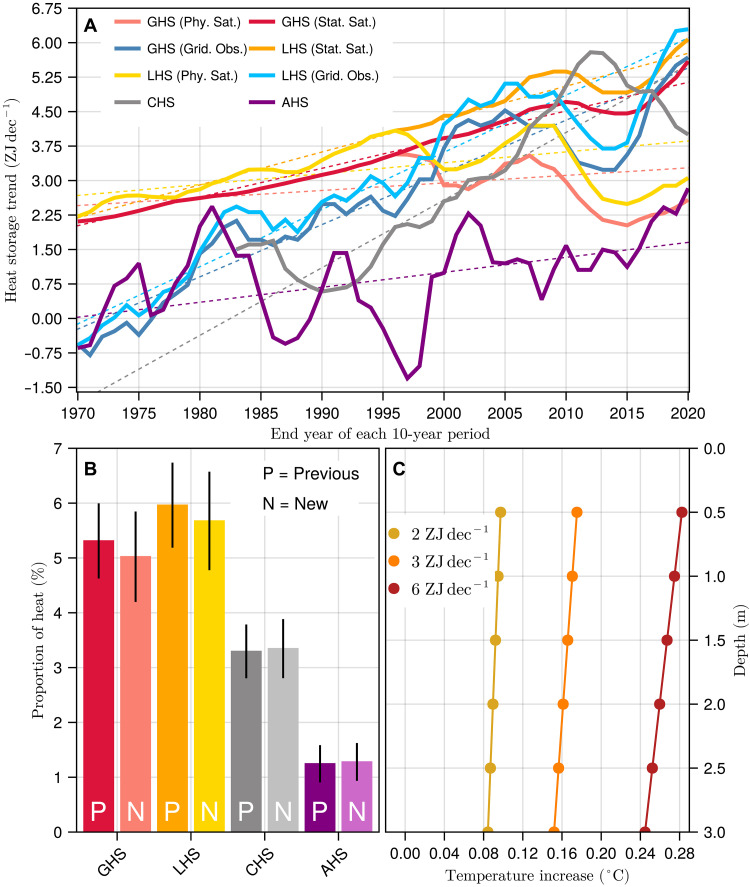
Heat uptake evolution and subsurface temperature increases. (**A**) Rate of change of heat storage in the ground, the land system (ground warming, permafrost thawing, and lake warming), the cryosphere, and the atmosphere for 10-year periods. (**B**) Proportion of heat stored in the same subsystems relative to the total heat in the Earth system. (**C**) Subsurface temperature warming for different GHS trends. GHS and LHS in (A) corresponds to estimates derived in this work and data from ref. (*27*), whereas cryosphere and atmosphere data were retrieved from ref. (*3*). Light colors and N letters in the bars of (B) correspond to estimates considering the values of GHS from the combination of satellite and geothermal data, whereas solid colors and P letters indicate results obtained with data from ref. (*3*). GHS, ground heat storage; LHS, land heat storage; CHS, cryosphere heat storage; AHS, atmosphere heat storage.

We assess the rate of change of the new and old GHS estimates together with cryosphere heat storage (CHC; the heat due to melting of ice caps, ice sheets, and glaciers) and atmosphere heat storage (AHC; heat stored in the warming of the atmosphere and the increase in humidity) from ref. (*3*). All components of the Earth heat inventory show increasing decadal rates of heating since the 1960s (Fig. 3A), which means that the components are gaining heat faster with time. That is, there is an acceleration in heat storage for all components that we measure as the trend in decadal rates of change (*38*), meaning that the trends in heat storage are larger in recent times than in the 1960s. The rate of ground heat uptake estimated from satellite and geothermal data in 1961 to 1970 is 2.109 ± 0.043 ZJ dec^−1^ (*P* < 0.01) for the physical and statistical reconciliation approaches, but in 2011 to 2020, the rate of heat uptake is larger for the physical estimate (2.58 ± 0.36 ZJ dec^−1^, *P* < 0.01) and the statistical estimate (5.60 ± 0.41 ZJ dec^−1^, *P* < 0.01). The land heat storage (LHS) in Fig. 3 corresponds to the combination of GHS, heat storage due to permafrost thawing, and heat storage due to lake warming. Permafrost heat storage was estimated in ref. (*39*) from the latent heat of fusion of water and the mass of permafrost thawed, with lake heat storage being derived from the warming of water in lakes and artificial reservoirs in ref. (*40*). The global GHS from satellite and geothermal data presents an acceleration in the rate of heat uptake of 0.16 ± 0.15 ZJ dec^−2^ (*P* < 0.01) according to the physical reconciliation approach and 0.624 ± 0.032 ZJ dec^−2^ (*P* < 0.01) according to the statistical approach. The updated LHS from ref. (*3*) with the GHS estimates derived from satellites and geothermal data achieves an acceleration term of 0.24 ± 0.15 ZJ dec^−2^ (*P* < 0.01) and 0.653 ± 0.056 ZJ dec^−2^ (*P* < 0.01) considering the physical and statistical approaches to derive GHS, respectively. The estimates using gridded meteorological observations to estimate GHS yield a larger acceleration term of 1.24 ± 0.14 ZJ dec^−2^ (*P* < 0.01). The rest of the Earth heat inventory components also present positive acceleration terms: The atmosphere achieves an acceleration of 0.33 ± 0.18 ZJ dec^−2^ (*P* < 0.01), with the cryosphere showing an acceleration of 1.47 ± 0.29 ZJ dec^−2^ (*P* < 0.01) since 1971, the second-largest acceleration after the ocean [16 ± 5 ZJ dec^−2^; ref. (*38*)]. Such acceleration in heat storage across the components of the Earth heat inventory is consistent with the acceleration detected in radiative forcing (*41*), sea level rise (*42*), and surface air temperature (*43*). Although the cryosphere presents a higher acceleration term than those of the land and the atmosphere, we find that the continental subsurface is still the second-largest term of the Earth heat inventory after the average of the physical and statistical estimates of GHS is included in the analysis (Fig. 3B), storing a 5.0 ± 0.8% of the total heat in the climate system (considering satellite and geothermal data combined), followed by the cryosphere (3.3 ± 0.5%) and the atmosphere (1.3 ± 0.4%).

The largest uncertainty of this analysis arises from the method used to reconcile heat storage estimates from geothermal and satellite data (fig. S1). We explore two reconciliation methods, a physical one, based on adding information about the preindustrial temperature climatology to the satellite temperature series, and a statistical one, based on adjusting the mean flux from the satellite estimates to have the same mean as the geothermal fluxes during the period covered by each satellite product. Both approaches relay on low-temporal resolution information form geothermal data to modify the annual-resolution series from satellites, introducing a source of uncertainty in the merging process. These uncertainties accumulate with time because the method used to derive GHFs takes into account previous temporal information for producing the flux datum at each time step (eq. S1). In the statistical approach, the mean of all satellite fluxes are forced to be the same, resulting in satellites providing temporal variability to the final heat storage estimate. The temperature climatology used in the physical approach to provide a preindustrial reference to the satellite products also presents uncertainties. The timing of the reference corresponds to approximately 1300 to 1700 CE, but there is a larger contribution of the conditions closer to 1700 CE to the average. Also, this temperature climatology refers to ground surface temperatures, whereas the satellite temperatures analyzed here are skin temperatures. Furthermore, the physical approach produces a GHS evolution inconsistent with other climate variables and with the statistical approach (fig. S1) as the rate of heat uptake decreases with time during a period in which the heat storage in the rest of components of the Earth heat inventory, the radiative flux, and the surface air temperature display an accelerating increase (*38*, *41*, *43*). Therefore, we can consider the estimates from the physical reconciliation approach as a lower limit for global GHS and the estimates obtained with the statistical reconciliation approach as an upper limit.

Different rates of ground heat uptake with time are translated into different rates of subsurface warming. For example, a GHS of 2 ZJ in a decade approximately leads to a warming of the global subsurface at 30 cm of depth of ~0.10°C (Fig. 3C). Nevertheless, an uptake rate of 6 ZJ in a decade, which remains plausible under an RCP4.5 scenario (*44*), leads to nearly triple the global subsurface warming, reaching 0.29°C at the same depth. The heat uptake is not spatially homogeneous with higher subsurface warming expected in temperate and polar regions of the Northern Hemisphere. Although these temperature increases seem small, soil carbon stored in the topsoils of almost all biomes around the world are reactive to changes in temperature (*45*), particularly temperate forests and permafrost soils (*12*, *13*, *46*). In addition, the stability of carbon stored in the first meter of soil is threatened by future soil temperature increases (*46*, *47*), with a projected global soil temperature increase between 2.3° and 4.5°C by the end of the 21st century under a range of future scenarios (*48*). The projected soil warming in permafrost areas is even higher, where soil temperatures could reach between 3.2° and 5.8°C above 1986 to 2005 levels (*48*). Ground heating in these permafrost regions may lead toward destabilizing the stored subsurface carbon and releasing active greenhouse gases, exacerbating the Earth energy imbalance (*49*, *50*).

## DISCUSSION

Although the heat storage by the terrestrial subsurface is the second-largest term of the Earth heat inventory, it is one of the least measured terms, and until recently it did not have a sustainable data source available for the future. Estimates from gridded surface air temperature and satellite LST products offer an indirect method for GHS monitoring but with important limitations. These approaches should include information about soil type, soil moisture, and land cover type, perhaps adapting the methods developed here for satellite data. Furthermore, the quality of heat storage estimates from both satellite and gridded observations still need to be assessed at regional and local scales. Geothermal data will continue to constitute the basis to estimate heat storage from satellite products for most of the 20th century and previous periods. Updating and expanding the geothermal database by retrieving subsurface temperature profiles in poorly sampled areas remains fundamental to obtain the full picture of long-term heat storage in the terrestrial subsurface. Additional direct observations of ground surface temperature and flux at meteorological stations and eddy-covariance towers are critical not only for estimating heat storage and evaluating indirect approaches but also for understanding the contribution of the thermal state of the shallow subsurface to land-atmosphere feedbacks governing extreme temperature events (*18*).

**Fig. 4.**
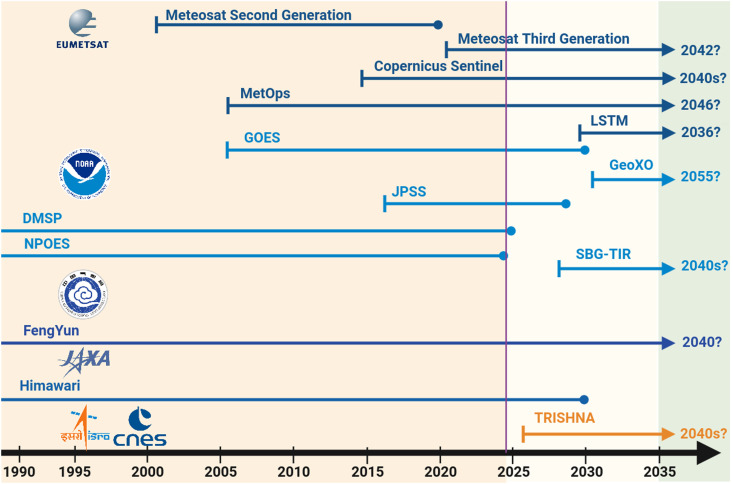
Expected life span of relevant current and future Earth observation missions. The included missions provide data to estimate GHS over the whole land surface from main meteorological and space agencies. Suitable satellite observations to derive GHS are virtually guaranteed for the next decade and possibly beyond the 2030s.

There are already established plans for monitoring all the ice-free terrestrial surface from satellite platforms in the future. Because satellite observations are routinely used by meteorological services around the world, the availability of geostationary and polar orbiting platforms equipped with different sensors is probably ensured for the next decade and beyond (Fig. 4). Furthermore, the life span of the LST group of the ESA-CCI, responsible for creating the satellite temperature products used here, is expected to operate until at least 2026 and possibly into the 2030s.

The long-term data availability and extensive spatial coverage of satellite products provide a clear and robust strategy for observing changes in GHS in the future, ensuring a comprehensive monitoring of the Earth’s heat inventory at both global and regional scales. Beyond the heat stored by the terrestrial subsurface, the remote sensing resources planned for the next decades should be exploited, together with modeling approaches, to aid in the determination of other components of the Earth heat inventory for which there are still insufficient or unsuitable observations, such as heat storage in permafrost and inland waters (*22*). Furthermore, information about the regional evolution of the heat storage in the ground, permafrost, and inland waters will also help to develop better land surface model components that are able to represent a more realistic Earth heat inventory (*28*, *44*), a persistent issue in climate model simulations (*26*).

## MATERIALS AND METHODS

### Eddy-covariance towers

Observations of ground surface temperatures and GHF at eddy-covariance towers were gathered to evaluate the satellite products and the ERA5-Land reanalysis. Measurements from FluxNet 2014 (*51*), the Integrated Carbon Observation System (ICOS) (*52*), and Ameriflux (*53*) databases are combined to maximize the temporal and spatial coverage of GHF measurements. These databases contain observations of multiple variables relevant for ecosystem science at a very fine temporal resolution (half-hourly and hourly), allowing to monitor the behavior of the carbon, hydrology, and energy cycles at hundreds of locations around the world. Monthly ground surface temperatures and GHF are derived for each eddy-covariance tower by aggregating daily averages. At least four measurements under daylight conditions and four measurements under nighttime conditions were imposed as a requirement to derive a daily average, and at least 24 days with nonmissing data are required to derive monthly averages.

### Land surface temperatures

Eight LST products (Fig. 1 and table S1) from the ESA-CCI (*54*) and NASA MODIS (*55*, *56*) are used to derive global estimates of GHF. Seven products were derived from infrared sensors: the Environmental satellite–Advanced Along-Track Scanning Radiometer (ENVISAT-AATSR), the merged InfraRed Climate Data Record (IRCDR), the InfraRed Merged Geostationary Platforms (IRMGP)GP, and the MODerate-resolution Imaging Spectroradiometer Aqua and Terra (MODIS-Aqua, MODIS-Terra, MOD10C3, and MYD11C3). An additional LST dataset based on the SSMI-SSMIS product was also used. Monthly estimates of LST are derived by computing the average of day and night observations from each satellite product.

The satellite LST products have been previously evaluated against in situ observations (*57*–*60*). Here, we complement these evaluations by comparing satellite products with observations from eddy-covariance towers and ERA5-Land data. All-day averages of satellite LSTs present lower unbiased root mean square errors (ubRMSEs) in comparison with ground surface temperatures from eddy-covariance towers than daytime and nighttime observations separately (fig. S2A). ERA5-Land temperatures present lower ubRMSEs than satellite products by ~1°C, but all satellite estimates of LST achieve median Pearson’s correlation coefficients higher than 0.9 (fig. S2B). These results, together with the evaluations in the literature, indicate that these temperature products represent well-enough surface conditions to be used for deriving GHFs. Estimates of GHF from satellite LSTs compare well against direct observations of GHF from eddy-covariance towers, reaching low root mean square errors and high correlation coefficients (fig. S1). In addition, satellite-based estimates have a similar performance to the heat fluxes from the state-of-the-art ERA5-Land reanalysis.

### Land cover type

Land cover data are retrieved from the ESA-CCI Land Cover project (*61*). Nevertheless, because FluxNet data are used as the evaluation reference in this analysis, the ESA-CCI Land cover data are reclassified to follow the categories of the International Geosphere-Biosphere Programme (*62*) (see table S2), which is the classification scheme used by FluxNet stations. The ESA-CCI Land Cover project provides data with a 299-m spatial resolution for the years 2009 to 2015, but only the land cover map for 2015 is used in this analysis as land cover does not change much at the global scale during the period of interest. Furthermore, a spatially interpolated version of the 2014 map is generated with the resolution of each LST product and of ERA5-Land reanalysis by applying the Largest Area Fraction remapping with the Climate Data Operators software (*63*) to facilitate the analysis. The largest area fraction algorithm retains the most common value of the original grid cells within each of the final, coarser grid cells.

### ERA5-Land data

The ERA5-Land reanalysis (*64*) produced by the European Center for Medium-Range Weather Forecast (ECMWF) is a land-focused enhancement of the fifth generation European ReAnalysis (ERA5) (*65*) developed by the same research center. ERA5-Land uses a downscaled version of the global ERA5 reanalysis as forcing of the CHTESSEL (Carbon Hydrology-Tiled ECMWF Scheme for Surface Exchanges over Land) (*66*), which is the same land surface model used in the ERA5 reanalysis. The finer resolution of ERA5-Land in comparison with ERA5 permits a better representation of topography and surface processes, and it includes a correction in several important atmospheric variables, such as relative and specific humidity, air temperature, and surface pressure to account for the different resolution of the land surface model. Ground (2.5 cm) and skin temperatures from ERA5-Land data are used as evaluation reference, as well as to obtain effective thermal properties for each land cover type.

### GHF estimates from satellite data

Estimates of GHF are derived from each satellite LST product (Fig. 1). These estimates are based on a solution of the one-dimensional heat diffusion equation considering a homogeneous land subsurface (*34*). The solution relates ground surface temperature and the thermal inertia of the subsurface with the corresponding GHF

$$\text{GHF}\left(t_{N}\right)=\frac{2\cdot \phi }{\sqrt{\pi }}\sum_{i=1}^{N-1}\frac{T_{i+1}-T_{i}}{t_{i+1}-t_{i}}\left(\sqrt{t_{N}-t_{i}}-\sqrt{t_{N}-t_{i+1}}\right)$$ (1)

where ϕ is the thermal inertia of the ground, $t_{i}$ is the *i*th time step, and $T_{i}$ is the ground surface temperature at the *i*th time step. An important advantage of this equation is that it can be applied to time series containing missing values, which maximizes the number of satellite grid cells with GHF estimates. Nevertheless, the main limitation for estimating GHF from satellite temperature data is the fact that satellite products represent skin temperatures instead of ground surface temperatures. Skin temperatures typically correspond to the temperature of the objects, vegetation, soil, or rocks that are viewed by the satellite sensors, whereas ground temperatures consist of the temperature below the surface at a certain depth, usually close to the surface. Both temperatures are thermally coupled over the long term, but different land covers and soil moisture conditions lead to different coupling levels (*67*, *68*). Therefore, an effective thermal inertia is considered here to account for the difference between skin and ground temperature. The effective thermal inertia is estimated by calibrating its value in Eq. 1 for each land cover using ERA5-Land reanalysis data. A linear regression analysis is performed between the GHF and the weighted skin temperature aggregation in Eq. 1, deriving the corresponding thermal inertia from the slope of the fitted line (*37*).

Because the period of reference of geothermal estimates of GHF corresponds to approximately 1300 to 1700, the global LST temporal series of each satellite product are modified by adding an extra time step containing the global ground surface temperature climatology for the mentioned period. Thereby, global GHF estimates from geothermal data and satellite products share the same reference conditions. This long-term climatology is estimated from the geothermal data following the methodology detailed in ref. (*69*).

Once the thermal inertia for all land covers is determined, Eq. 1 is applied to the modified LSTs from the satellite products to derive the global GHF evolution. In this case, the global thermal inertia is the weighted mean of each land cover type, with the number of grid cells for each land cover serving as the weights. Then, Eq. 1 is applied to the LST of each grid cell to obtain a spatial product of GHF for each satellite product, accounting for the land cover type of the grid cell.

### Heat fluxes from ERA5-Land data

The ERA5-Land reanalysis does not provide GHF data. Therefore, heat flux is derived using ground surface temperature and soil moisture data at the first layer of the reanalysis. Because ERA5-Land represents the evolution of ground surface temperature, there is no need of considering an effective thermal inertia at each grid cell. Instead, the thermal inertia is estimated considering soil moisture from ERA5-Land, along with sand and clay data from the SoilGrids dataset (*70*). This thermal inertia and the ground surface temperature in ERA5-Land are then used to drive Eq. 1.

### Subsurface temperature warming

A purely conductive forward model is used to derive changes in subsurface temperatures for different ground heating scenarios (*71*). This model considers a homogeneous, semi-infinite subsurface without internal sources of heat, and it is able to propagate changes in surface temperature through the subsurface as

$$T\left(z\right)=\sum_{k=0}^{N}\Delta T_{k}\left[\text{erfc}\left(\frac{z}{2\sqrt{\alpha \cdot t_{k}}}\right)-\text{erfc}\left(\frac{z}{2\sqrt{\alpha \cdot t_{k-1}}}\right)\right]$$ (2)

where T(z) is the subsurface temperature (K), ΔT is the surface temperature anomaly (K), z is the depth (m), α is the thermal diffusivity (m^2^ s^−1^), and *t* is the time before present. In this study, a constant thermal diffusivity of 1.0 × 10^−6^ m^2^ s^−1^ is used.

The ground heating level for each scenario in Fig. 3C arises from integrating the subsurface temperature profile generated by forcing Eq. 2 with different surface temperatures over a 9-year period. These surface signals consist in a temperature series increasing with time according to a prescribed rate. This rate is adjusted to obtain the heating level indicated in each scenario.
