# Supplementary material for: Robust increase in observed heat storage by the global subsurface
Source: Sci Adv. 2025 Nov 12;11(46):eadw9958. doi: 10.1126/sciadv.adw9958 (PMC12609063; doi:10.1126/sciadv.adw9958)
Supplement: Supplementary file 1 — Figs. S1 and S2 Tables S1 and S2 [file sciadv.adw9958_sm.pdf]

Science Advances
AAAS

Supplementary Materials for

Robust increase in observed heat storage by the global subsurface

Francisco José Cuesta-Valero *et al.*

Corresponding author: Francisco José Cuesta-Valero, francisco-jose.cuesta-valero@ufz.de;
Jian Peng, jian.peng@ufz.de

Sci. Adv. **11**, eadw9958 (2025)
DOI: 10.1126/sciadv.adw9958

This PDF file includes:

Figs. S1 and S2
Tables S1 and S2

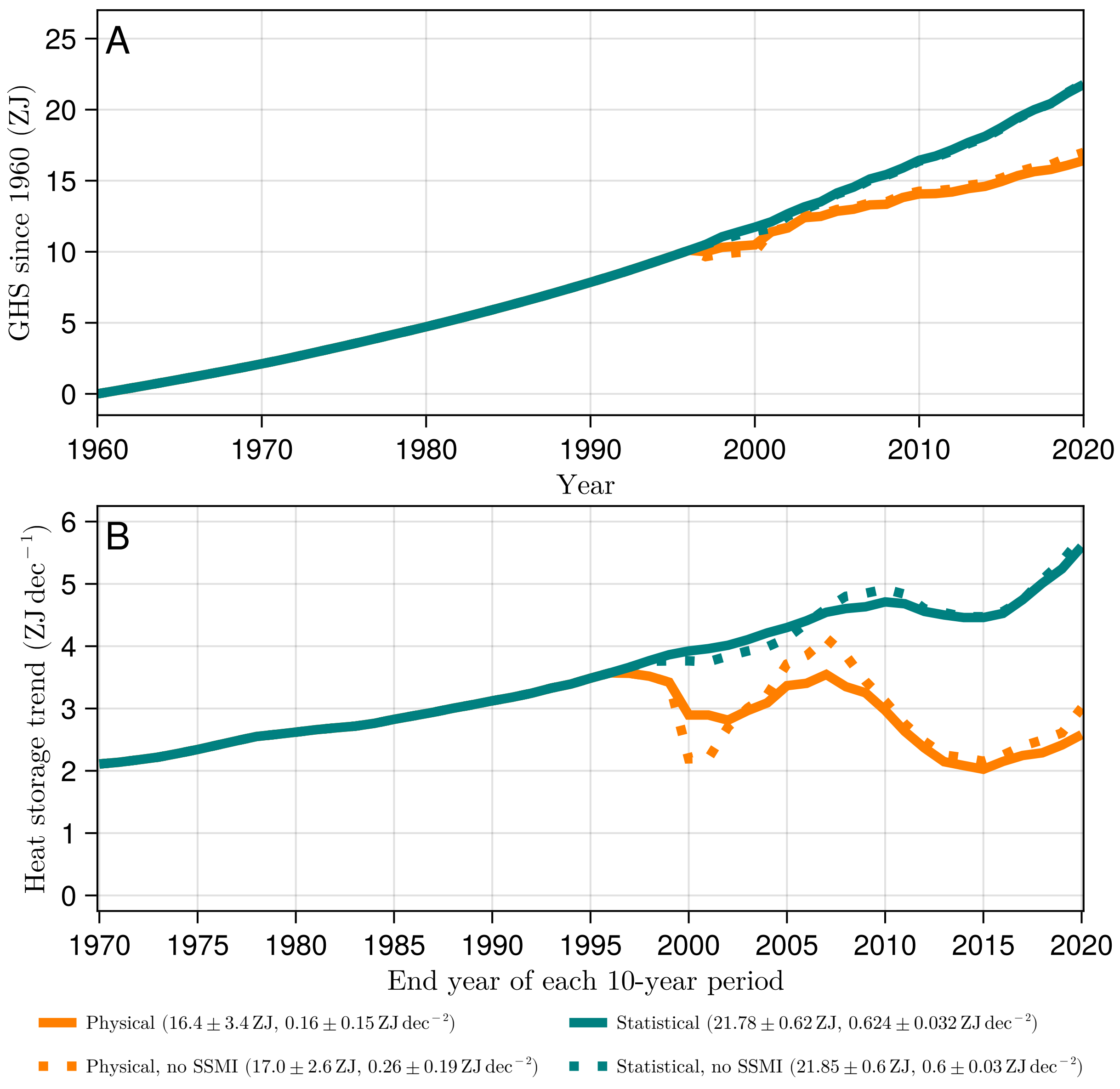

Figure S1: **Comparison between the physical and statistical approaches to reconcile heat storage estimates from geothermal and satellite data.** (**A**) Temporal evolution of heat storage using both methods. (**B**) Rate of heat uptake in the ground for 10-year periods. Dotted lines indicate estimates without considering data from the SSMI-SSMIS product in the analysis. Heat storage since 1960 and the acceleration, estimated as the trend of the rate of heat uptake for each method, are indicated in brackets.

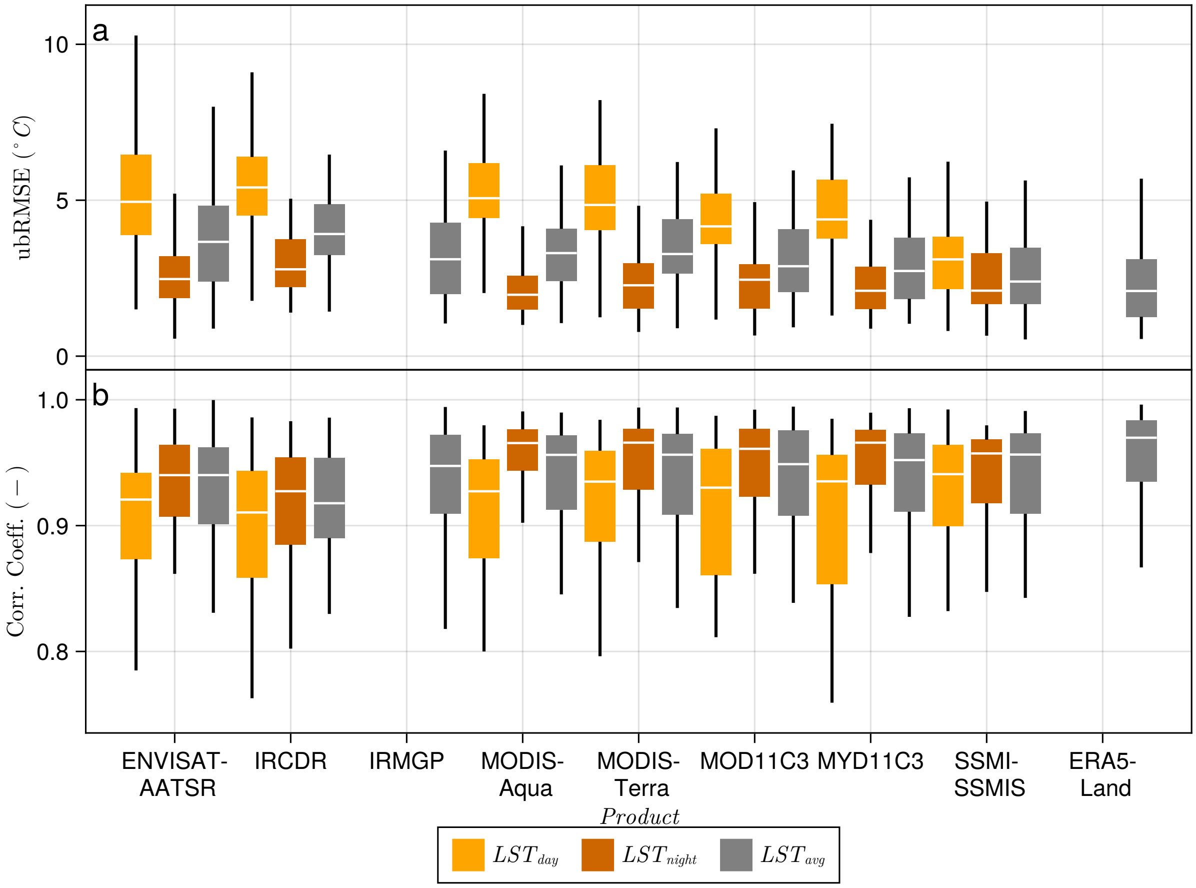

Figure S2: **Evaluation of surface temperatures from ESA-CCI satellite products and ERA5-Land reanalysis against observations from Eddy-covariance towers.** (**A**) Unbiased root mean squared errors, and (**B**) correlation coefficients. Golden boxes indicate results for daytime conditions, orange boxes indicate results for nighttime conditions, gray boxes indicate results for all-day conditions. The top and the bottom of the boxes represent the 75 % and 25 % percentiles of the distribution, with the median indicated by the white central line. Whiskers represent values within 1.5 times the interquartile range.

Table S1: **Land surface temperature products considered for deriving ground heat flux.** The name and version of the product are indicated in the first two columns, the sensors used to derive the products and the corresponding satellite platforms are indicated in the third and fourth columns, respectively, with the spatial resolution, and the temporal period indicated in the fifth and sixth columns, respectively.

Product	Version	Instrument	Satellite	Spatial resolution	Time period
ENVISAT-AATSR	3.00	AATSR	Envisat	1 km	August, 2002 to March, 2012
IRCDR	2.00	ATSR-2, AATSR, MODIS, SLSTR	ERS-2, Envisat, EOS Terra, Sentinel-3A	1 km	August, 1995 to December, 2020
IRMGP	1.00	SEVIRI, IMAGER, ABI, JAMI, ATSR-2, AATSR, MODIS, SLSTR	MSGx, GOESx, MTSATx, ERS-2, Envisat, EOS Terra, EOS Aqua, Sentinel-3x	5 km	January, 2009 to December 2020
MODIS-Terra	3.00	MODIS	EOS Terra	1 km	March, 2000 to December, 2018
MODIS-Aqua	3.00	MODIS	EOS Aqua	1 km	July, 2002 to December, 2018
MOD11C3	6.10	MODIS	EOS Terra	5 km	February, 2000 to December, 2020
MYD11C3	6.10	MODIS	EOS Aqua	5 km	July, 2002 to December, 2020
SSMI-SSMIS	2.33	SSM/I, SSMIS	DMSP	25 km	January, 1996 to December, 2020

Table S2: **Land cover type classification.** IGBP category (first column) and corresponding categories in the ESA CCI Land cover product (second column).

IGBP	ESA CCI Land cover
No data	No data
Evergreen needleleaf forest	Tree cover needleleaved evergreen closed to open (¿15%)
	Tree cover needleleaved evergreen closed (¿40%)
	Tree cover needleleaved evergreen open (15-40%)
Evergreen broadleaf forest	Tree cover broadleaved evergreen closed to open
Decidiuous needleleaf forest	Tree cover needleleaved deciduous closed to open (¿15%)
	Tree cover needleleaved deciduous closed (¿40%)
	Tree cover needleleaved deciduous open (15-40%)
Decidiuous broadleaf forest	Tree cover broadleaved deciduous closed to open (¿15%)
	Tree cover broadleaved deciduous closed (¿40%)
Mixed forest	Tree cover mixed leaf type (broadleaved and needleleaved)
Closed shrublands	Shrubland
	Shrubland evergreen
Open shrublands	Mosaic herbaceous cover (¿50%) / tree and shrub (¡50%)
	Sparse vegetation (tree shrub herbaceous cover) (¡15%)
Woody savannas	Mosaic tree and shrub (¿50%) / herbaceous cover (¡50%)
	Shrubland deciduous
Savannas	Tree cover broadleaved deciduous open (15-40%)
	Sparse tree (¡15%)
	Sparse shrub (¡15%)
Grasslands	Grasslands
Permanent wetlands	Tree cover flooded fresh or brakish water
	Tree cover flooded saline water
	Shrub or herbaceous cover flooded fresh/saline/brakish water
Croplands	Cropland rainfed
	Cropland herbaceous cover
	Cropland tree or shrub cover
	Cropland irrigated or post-flooding
Urban and built-up lands	Urban areas
Cropland/natural vegetation mosaics	Mosaic cropland (¿50%) / natural vegetation (tree shrub herbaceous cover) (¡50%)
	Mosaic natural vegetation (tree shrub herbaceous cover) (¿50%) / cropland (¡50%)
Snow and ice	Permanent snow and ice
Barren	Sparse herbaceous cover
	Bare areas
	Consolidated bare areas
	Unconsolidated bare areas
Water bodies	Water bodies
Tundra	Lichens and mosses
